# Supplementary material for: A Global Screen for Assembly State Changes of the Mitotic Proteome by SEC-SWATH-MS
Source: Cell Syst. 2020 Feb 26;10(2):133–155.e6. doi: 10.1016/j.cels.2020.01.001 (PMC7042714; doi:10.1016/j.cels.2020.01.001)

Q9Y6D9 | MD1L1_HUMAN | MAD1L1 MAD1 TXBP181
Monomer MW [kDa]: 83.067 Monomer expected elution fraction: 42

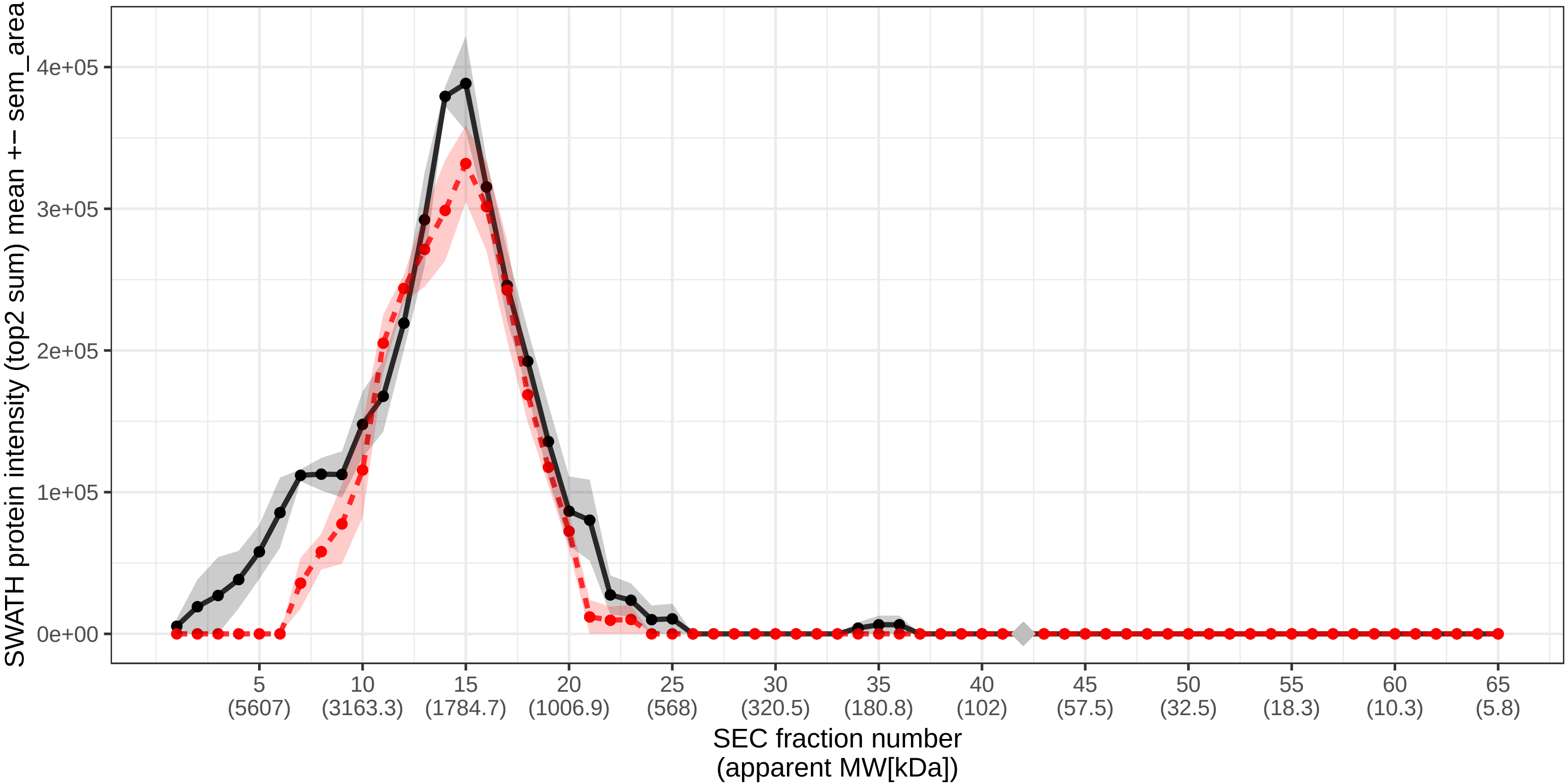

Supplement: Data S1. SEC-SWATH-MS Protein Chromatograms, Related to Figure 1 [file mmc6.zip › SECchrom_Q9Y6D9_MD1L1_HUMAN_MAD1L1_MAD1_TXBP181.pdf]
